# Functional properties of lactic acid bacteria isolated from Tilapia (*Oreochromis niloticus*) in Ivory Coast

**DOI:** 10.1186/s12866-023-02899-6

**Published:** 2023-05-25

**Authors:** Wahauwouélé Hermann Coulibaly, N’goran Richard Kouadio, Fatoumata Camara, Camelia Diguță, Florentina Matei

**Affiliations:** 1 Biotechnology and Food Microbiology Laboratory, Food Science and Technology, Formation and Research Unit, University Nangui Abrogoua, 02 BP 801 Abidjan 02, Abidjan, Côte d’Ivoire; 2 Nutrition and Food Safety Laboratory, Food Science and Technology, Formation and Research Unit, University Nangui Abrogoua, 02 BP 801 Abidjan 02, Abidjan, Côte d’Ivoire; 3 Applied Microbiology Laboratory, Faculty of Biotechnologies, University of Agronomic Sciences and Veterinary Medicine Bucharest, 59 Mărăsti Blvd, Bucharest, 011464 Romania

**Keywords:** Tilapia fish, *Pediococcus acidilactici*, *Pediococcus pentosaceus*, *Lactobacillus plantarum*, Probiotics, Aquaculture

## Abstract

**Background:**

Probiotics have recently been applied in aquaculture as eco-friendly alternatives to antibiotics to improve fish health, simultaneously with the increase of production parameters. The present study aimed to investigate the functional potential of lactic acid bacteria (LAB) isolated from the gut of Tilapia (*Oreochromis niloticus*) originating from the aquaculture farm of Oceanologic Research Center in Ivory Coast.

**Results:**

Twelve LAB strains were identified by 16 S rDNA gene sequence homology analysis belonging to two genera *Pediococcus* (*P. acidilactici* and *P. pentosaceus*) and *Lactobacillus* (*L. plantarum*) with a predominance of *P. acidilactici*. Several aspects including functional, storage, and safety characteristics were taken into consideration in the selection process of the native LAB isolates as potential probiotics. All LAB isolates showed high antagonistic activity against bacterial pathogens like *Escherichia coli, Klebsiella pneumoniae, Pseudomonas aeruginosa, Proteus mirabilis*, and *Staphylococcus aureus*. In addition, the LAB isolates exhibited different degrees of cell surface hydrophobicity in the presence of hexane, xylene, and chloroform as solvents and a good ability to form biofilm. The strong antioxidant activity expressed through the DPPH scavenging capacity of LAB intact cells and their cell-free supernatants was detected. LAB strains survived between 34.18% and 49.9% when exposed to low pH (1.5) and pepsin for 3 h. In presence of 0.3% bile salts, the growth rate ranged from 0.92 to 21.46%. Antibiotic susceptibility pattern of LAB isolates showed sensitivity or intermediate resistance to amoxicillin, cephalothin, chloramphenicol, imipenem, kanamycin, penicillin, rifampicin, streptomycin, tetracycline and resistance to oxacillin, gentamicin, and ciprofloxacin. No significant difference in antibiotic susceptibility pattern was observed between *P. acidilactici* and *P. pentosaceus* strains. The non-hemolytic activity was detected. Following the analysis of the enzyme profile, the ability of LAB isolates to produce either lipase or β-galactosidase or both enzymes was highlighted. Furthermore, the efficacy of cryoprotective agents was proved to be isolate-dependent, with LAB isolates having a high affinity for D-sorbitol and sucrose.

**Conclusion:**

The explored LAB strains inhibited the growth of pathogens and survived after exposure to simulated gastrointestinal tract conditions. The safety and preservative properties are desirable attributes of these new probiotic strains hence recommended for future food and feed applications.

## Background

In West Africa, intensive and semi-intensive systems of aquaculture farming remain the most common among fish farmers [1, 2]. Bamba et al. [3]; Gabriel et al. [4] and Crentsil and Ukpong [5] reported a massive use of agro-industrial by-products of plant origin (wheat bran, corn bran, rice bran, low rice flour) at a lower cost as feed for fish farming on most fish farms in sub-Saharan Africa. However, these agro-industrial by-products have a low protein, nutritional and immune contribution [6]. Furthermore, an inadequate application of antibiotics to boost fish production could lead to adverse disorders such as an imbalance in the gut microbiota, poisoning, immunity reduction as well as predisposition to the development of diseases [7–9]. Moreover, using antibiotics could be a potential risk to the health of consumers since the vast majority of antibiotics used are the same used to treat human infections [9]. Currently, probiotics are intensively promoted as healthy alternatives for sustainable aquaculture [10–14].

According to the World Health Organization (WHO) and the Food and Agriculture Organization (2014), probiotics were defined as “live microorganisms that, when administered in adequate amounts, confer a health benefit on the host” [15, 16]. Lactic acid bacteria (mainly *Lactobacillus sp., Bifidobacterium* sp. and *Pediococcus* sp.) [17–20], *Bacillu*s sp. [20, 21] and a few yeasts (mainly *Saccharomyces boulardii* and *S. cerevisiae*) [22–24] are intensively studied as probiotics to improve aquatic life health and performance of fish. However, it was found that the probiotic bacteria isolated from other hosts used in aquaculture do not colonize efficiently the fish gut as the native (indigenous) probiotics [25–27]. In their study, Boutin et al. [26] reported that native probiotic strains are a better choice than exogenous probiotics which could cause the homeostatic disturbance of the fish microbiota. Recently, research has focused more on host-associated microorganisms as a source of probiotics, due to the fact that the health beneficial effects could be species-specific, as well as that they adapt much more easily to the aquatic environment (e.g. salinity, temperature) [28–34]. Microorganisms with potential use as probiotics have been isolated from the gastrointestinal tract of Atlantic cod (*Gadus morhua*) [31], common carp [32], giant freshwater prawn (*Macrobrachium rosenbergii*) [33], rainbow trout (*Oncorhynchus mykiss*) [34], Nile Tilapia (*Oreochromis niloticus*) [29, 30, 35], Mediterranean trout (*Salmo macrostigma*) [36]. The choice of inappropriate microbes could have been the cause of the negative results observed in probiotic research [27, 37–39]. Different functionality, safety, and storage criteria have been established to investigate the microbial strains with probiotic potential, thus allowing the screening of the most promising strains [27, 37–39]. Generally, the criteria for the selection of probiotics are highlighted as antibacterial activity, antibiotic susceptibility, simulation of gastrointestinal conditions [40–46] biofilm-forming ability [47–50], hemolytic activity [51], hydrophobicity [52], antioxidant activity [53, 54], and enzymes production [36, 55, 56].

Currently, many commercial probiotics which contain one or more live microorganisms are introduced in fish farming industries mainly to improve the growth performance and boost the health of fish [57–60]. According to the study of Nimrat and Vuthiphandchai [57], none of the 12 commercial probiotics used in marine shrimp culture in Thailand did offer correct informations about the composition or number of micro-organisms or qualitative extracellular enzymes described on the labels. Furthermore, none of the commercial probiotics could inhibit the growth of the shrimp pathogen *V. harveyi* [57].

Several studies have proven some beneficial effects linked to the administration of native probiotics on fish species, including high feed conversion efficiency, supply of nutrients and enzymatic input to digestion, increased growth performance and stimulation of the immune system [61–72]. overall suggesting that native probiotics could be relevant alternatives to antibiotics to control emerging fish diseases, increase stress resistance and improve water quality [61–72]. Fish production may therefore be improved by using indigenous probiotics for the sustainable development of African aquaculture [73].

Thus, the current study aimed to investigate the lactic acid bacterial (LAB) strains isolated from the gut of Tilapia (*Oreochromis niloticu*s) as potential probiotics, by addressing their functional properties (antibacterial activity, biofilm-forming ability, simulation of gastrointestinal conditions, hydrophobicity, antioxidant, and enzymatic activities), safety (antibiotic sensibility and hemolytic activity) and storage (freeze-drying survival).

## Results

### Molecular identification of LAB isolates

The LAB strains included in the study (Table 1) were isolated from the intestine of Tilapia (*Oreochromis niloticus*) originating from the aquaculture farm of the Oceanologic Research Center in Ivory Coast. The full-length 16 S rDNA genes of all the LAB isolates were sequenced to identify them at the species level. A BLAST search of the 16 S rDNA gene sequences obtained was performed at NCBI and revealed high similarity values to many bacterial 16 S rDNA sequences deposited in the NCBI database. LAB strains identified belonged to two genera *Pediococcus* and *Lactobacillus*. The partial 16 S rDNA gene sequences of nine LAB strains (LB45, LB98, LB100, LB137, LB143, LB156, LB166, LB187, and LB194) were identified as *P. acidilactici* (showed 96.38 − 98.21% homology to the GenBank sequences). The other two LAB strains (LB82 and LB195) were identified as *P. pentosaceus* (97.43-99% homology to GenBank sequences). The LB96 strain had 97.66% homology with the known *Lactobacillus plantarum* sequences. The partial 16 S rDNA sequences of LAB strains were deposited in the NCBI database (the accession numbers are listed in Table 1).

**Table 1:** LAB strains accession number

Strains	Accession number
*Pediococcus acidilactici* LB100	ON141894
*P. acidilactici* LB98	ON141895
*P. acidilactici* LB187	ON141896
*P. acidilactici* LB194	ON141897
*P. acidilactici* LB166	ON141898
*P. acidilactici* LB156	ON141899
*P. acidilactici* LB143	ON141900
*P. acidilactici* LB45	ON141901
*P. acidilactici* LB137	ON141902
*P. pentosaceus* LB195	ON141903
*P. pentosaceus* LB82	ON141904
*Lactobacillus plantarum* LB96	ON141905

**Fig. 1:**
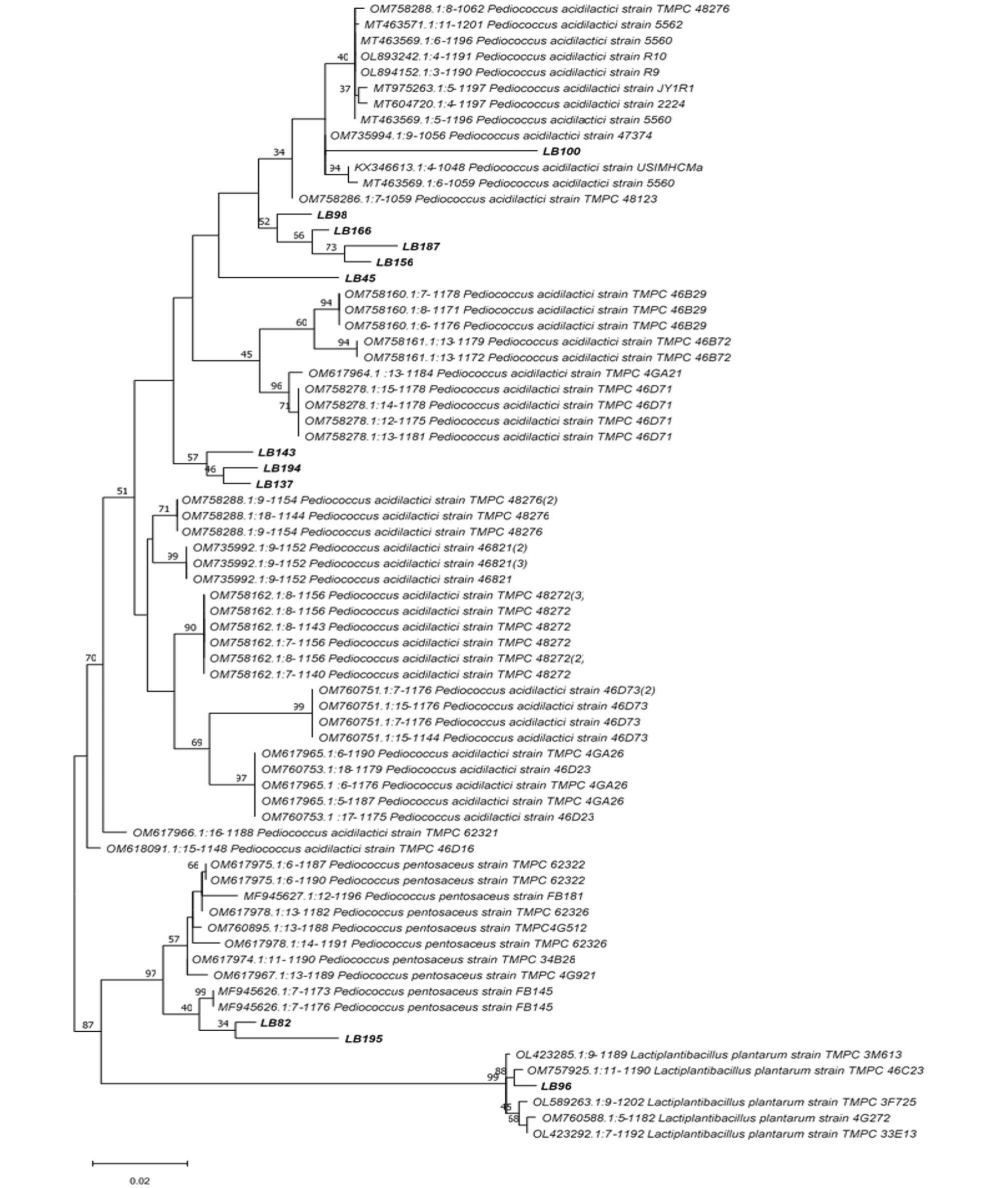
The phylogenetic tree showing the relative position of LAB isolates as inferred by the neighbour-joining method with 16 S rDNA gene sequences

The phylogenetic tree revealed the existence of several groups of LAB species (Fig. 1). Thus, the *L. plantarum* LB96 (ON141905) was related to *Lactobacillus plantarum* TMPC 3M613 strain (OM757925), supporting a bootstrap value of 88%. *P. pentosaceus* LB82 (ON141904) and LB195 (ON141903) were related to *P. pentosaceus* FB 145 strain (MF945626) with a bootstrap score of 40%. *P. acidilactici* strains (LB45, LB98, LB100, LB137, LB143, LB156, LB166, LB187, LB194) were related to different *P. acidilactici* strains.

### Functional properties of LAB isolates

#### Antibacterial activity of the cell-free supernatants (CFS) from the LAB strains

Antibacterial activity against pathogenic bacteria was considered an important criterion for the selection of probiotics. In this research, the antibacterial activity of cell-free supernatants (CFS) from the LAB isolates was assessed against five pathogens including *E. coli, K. pneumoniae, P. mirabilis, P. aeruginosa*, and *S. aureus* using agar well diffusion method. The value of the inhibition zone diameter expressed in mm is summarized in Table 2. The LAB isolates showed statistically significant (p < 0.05) inhibition rates regardless of the pathogen. Our results showed that the CFSs obtained from all LAB strains exhibited good antibacterial activity against tested pathogenic bacteria. Moreover, CFS from the LB143 strain seems to exert the highest inhibition effect against *S. aureus*, *P. mirabilis*, and *K. pneumoniae.* A similar inhibitory activity against *P. aeruginosa* and *E. coli* was observed for CFS from LB195.

**Table 2:** Antibacterial activity of LAB strains against pathogenic microorganisms

Isolates	*P. aeruginosa* ATCC 27853	*E. coli* ATCC 25922	* S. aureus* ATCC 25913	P. *mirabilis* JCM 1669	* K.* *pneumoniae* ATCC 43816
LB45	+ +	+ +	+ +	+ +	+ +
LB82	+ + +	+ +	+ +	+ +	+ +
LB96	+ +	+ +	+ +	+ +	+ +
LB98	+ +	+ + +	+ +	+ +	+ +
LB100	+ +	+ +	+ +	+ +	+ +
LB137	+ +	+ + +	+ +	+ +	+ +
LB143	+ +	+ +	+ + +	+ + +	+ + +
LB156	+ +	+ + +	+ +	+ +	+ +
LB166	+ +	+ +	+ +	+ +	+ +
LB187	+ +	+ +	+ +	+ +	+ + +
LB194	+ +	+ +	+ +	+ +	+ +
LB195	+ + +	+ + +	+ +	+ +	+ +

(+): 1–5 mm ; (+ +): 6–17 mm ; (+ + +): 18–29 mm

#### Ability to form a biofilm

All LAB isolates had a high capacity to form a biofilm. The absorbance values were higher than 0.5, ranging from 0.928 ± 00 (LB96) to 3.211 ± 0.01 (LB143). The statistical analyses showed a significant difference (p < 0.05) between the isolates (Table 3).

#### Hydrophobicity

The hydrophobicity test was carried out in the presence of hexane, xylene, and chloroform as solvents. The results revealed that hydrophobicity rates were 1.53 ± 0.1 and 16.30 ± 0.4% in the presence of hexane for isolates LB195 and LB156, respectively. In the presence of xylene as the solvent, isolate LB137 manifested the highest hydrophobicity (51.10 ± 0.8%), while the lowest was observed at isolate LB82 (1.17 ± 0.8%). The use of chloroform as solvent showed hydrophobicity values that ranged from 9.4 ± 0.14% (LB96) to 87.2 ± 0.14% (LB166). Furthermore, statistical analyses showed a significant difference (p < 0.05) between isolates for the same solvent and between different solvents (Table 3).

#### Antioxidant activity

In general, antioxidant activity levels were statistically significant (p < 0.05) for both supernatants and LAB intact cells. Overall, the antioxidant activity levels observed in the supernatants were higher than those in the intact cells (Table 3). However, for isolates LB96, LB137, and LB195 antioxidant activity values were highest in intact cells than in the supernatant with 87.28 ± 0.40, 70.40 ± 0.57%, and 57.19 ± 0.27%, respectively (Table 3). For each sample (supernatant and intact cells), statistical analyses showed a significant (p < 0.05) difference between the isolates.

**Table 3:** Ability to form a biofilm (AFB), antioxidant activity (AA), and hydrophobicity (H) of LAB isolates

Isolates	AFB	Antioxidant activity (AA) (%)		Hydrophobicity (H) (%)		
		Supernatants	Intact cells	Hexane	Xylene	Chloroform
LB 45	1.425 ± 0.04^a^	90.23 ± 0.32^a^	36.74 ± 0.05^b^	10.94 ± 0.01^a^	7.23 ± 0.1^b^	15.85 ± 0.32^c^
LB 82	2.886 ± 0.002^b^	51.92 ± 0.03^a^	49.95 ± 1.34^a^	9.96 ± 0.6^a^	1.17 ± 0.8^b^	51.9 ± 0^c^
LB 96	0.928 ± 0^c^	61.18 ± 0.25^b^	87.28 ± 0.40^a^	12.92 ± 1^a^	10.83 ± 0.3^ab^	9.4 ± 0.14^b^
LB 98	2.843 ± 0.06^b^	74.29 ± 0.12^a^	50.34 ± 0.03^b^	9.40 ± 0.2^a^	3.48 ± 0.4^b^	11.05 ± 0.17^ac^
LB 100	2.722 ± 0.03^b^	84.06 ± 0.08^a^	39.52 ± 0.73^b^	11.82 ± 2.1^a^	8.87 ± 0.6^b^	20.58 ± 0.29^c^
LB 137	1.616 ± 0.05^d^	67.86 ± 0.08^b^	70.40 ± 0.57^a^	7.38 ± 1.3^a^	51.10 ± 0.8^b^	10.49 ± 0.06^c^
LB 143	3.211 ± 0.01^e^	75.57 ± 0.81^a^	73.28 ± 0.38^a^	9.05 ± 0.4^a^	4.97 ± 0.2^b^	22.78 ± 0.07^b^
LB 156	1.489 ± 0^d^	64.52 ± 0.03^a^	44.19 ± 0.26^b^	16.30 ± 0.4^a^	8.31 ± 0.02^b^	20.47 ± 0^c^
LB 166	3.048 ± 0^e^	63.85 ± 5.4^a^	54.96 ± 1.36^a^	15.55 ± 0.01^a^	6.99 ± 0.03^b^	87.2 ± 0.14^c^
LB 187	1.362 ± 0.06^d^	57.84 ± 1.18^a^	34.06 ± 0.08^b^	11.52 ± 0.8^a^	7.62 ± 0.5^b^	23.18 ± 0.25^c^
LB 194	2.710 ± 0.007^b^	76.60 ± 0.85^a^	54.02 ± 0.02^b^	11.13 ± 0.3^a^	20.84 ± 1.2^b^	22.68 ± 0.9b^c^
LB 195	3.046 ± 0.01^e^	50.64 ± 0.05^b^	57.19 ± 0.27^a^	1.53 ± 0.1^a^	4.21 ± 0.6^b^	73.54 ± 0.85^c^

(-) Negative to hemolytic activity; Values expressed as mean ± standard deviation for three independent measurements.

AFB: Mean values with the same letter in a column were not significantly different (p > 0.05)

AA: Mean values with the same letter in a line were not significantly different (p > 0.05)

H: Mean values with the same letter in a line were not significantly different (p > 0.05)

#### Tolerance to bile salts and resistance to pepsin and acid pH of LAB isolates

**Fig. 2:**
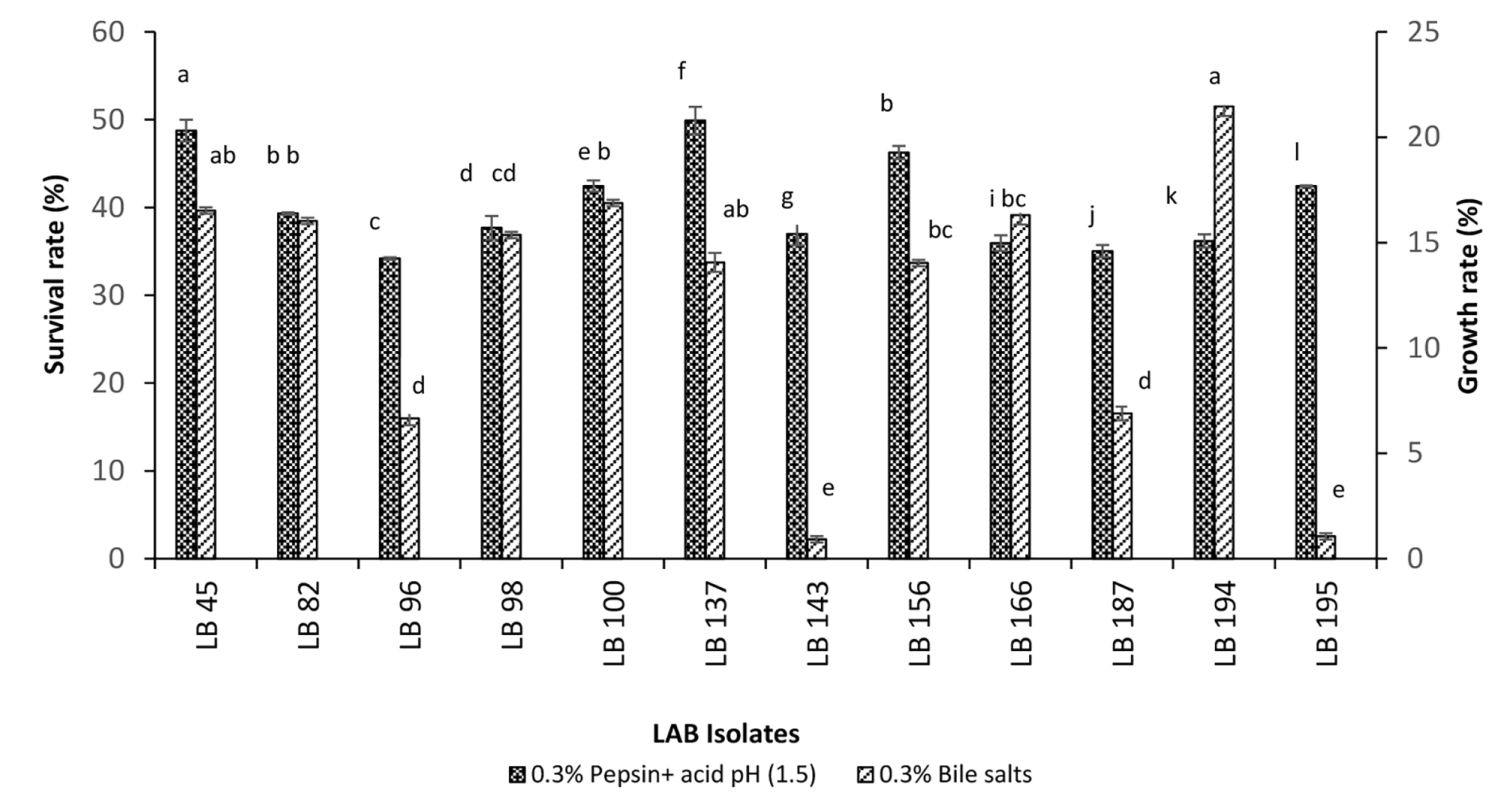
Growth rate and survival rate of LAB isolates in the presence of 0.3% bile salts and 0.3% pepsin and pH 1.5 respectively

A critical step toward the selection of probiotic strains was to survive conditions that mimic the gastrointestinal tract. The LAB isolates bile salts tolerance, as well as the resistance in the presence of pepsin and acid pH evolution tests are shown in Fig. 2. Generally, an adaptation of all LAB isolates was observed after 4 h of exposure to bile salts characterized by cell growth, while exposition after 3 h to 0.3% pepsin and acid pH (1.5) was marked with a decrease in bacterial load. Based on growth rates, three profiles were observed. The most significant growth (p < 0.05) was observed for isolate LB 194, with a growth rate of 27.33%, followed by isolates LB45, LB82, LB98, LB100, and LB166 (growth rate between 15.36 and 16.88%). The least growth rates were observed in isolates LB 143 and LB 195 (0.92 and 1.06%). The growth rates showed a significant difference (p < 0.05) between the three profiles.

Despite the overall decrease in bacterial load, the level of resistance to pepsin and acid pH (1.5) was reflected by a survival rate ranging from 34.18 to 49.9%. The highest survival rate was obtained for the LB166 isolate which was significantly different (p < 0.05), while the lowest rate was observed in the LB96 isolate.

Each value represents the mean value ± standard deviation (SD) (n = 3). Bars with different lower-case letters denoted significantly different (p < 0.05).

In vitro **investigation of enzymatic activities of LAB isolates**.

To detect amylase, protease, lipase, and β-galactosidase activities the LAB isolates were inoculated into selective media for each enzyme. Our results revealed that the nine LAB strains tested were positive for lipases (pink-orange colony under UV 352 nm) and β-galactosidase (green colony) (Table 4). LB 143 did not exhibit lipase activity, while LB 137 and LB 156 did not exhibit β-galactosidase activity. No activity was detected for the amylases, cellulases, and proteases, respectively (Table 4).

**Table 4:** Hydrolytic enzyme profile of the LAB isolates

Isolates	Enzymatic activities				
	Amylolytic	Cellulolytic	Lipolytic	Proteolytic	β-galactosidase
LB 45	-	-	+	-	+
LB 82	-	-	+	-	+
LB 96	-	-	+	-	+
LB 98	-	-	+	-	+
LB 100	-	-	+	-	+
LB 137	-	-	+	-	-
LB 143	-	-	-	-	+
LB 156	-	-	+	-	-
LB 166	-	-	+	-	+
LB 187	-	-	+	-	+
LB 194	-	-	+	-	+
LB 195	-	-	+	-	+

(+) and (-) indicate the presence and absence of the enzymatic activities

### Safety properties of LAB isolates

#### Antibiotic susceptibility

The antibiotic susceptibility of the LAB isolates was tested using the antibiotic disc diffusion method on MRS agar plates. A total of 12 antibiotics were included in the assay: gentamicin, chloramphenicol, kanamycin, streptomycin, tetracycline as inhibitors of protein synthesis, amoxicillin, cephalothin, oxacillin, penicillin, imipenem as inhibitors of cell wall synthesis, ciprofloxacin as inhibitors of DNA replication and rifampicin as inhibitors of nucleic acids synthesis. All LAB isolates showed variations in antibiotic susceptibility to 9 out of the 12 antibiotics and also showed multidrug resistance for oxacillin, gentamicin, and ciprofloxacin (Table 5). No significant difference in antibiotic susceptibility profile was observed between *P. acidilactici* and *P. pentosaceus* strains.

**Table 5:** Antibiotic susceptibility pattern of LAB isolates

Isolates	AML	OX	C	PEN	CN	TE	KAN	CIP	STR	RAM	IPM	GM
**LB45**	25 S	0R	28 S	27 S	11R	26 S	21 S	14R	30 S	35 S	26 S	22 S
**LB82**	28 S	0R	30 S	30 S	7R	24 S	25 S	10R	20 S	37 S	37 S	30 S
**LB96**	30 S	0R	29 S	30 S	14R	25 S	20 S	0R	25 S	32 S	25 S	27 S
**LB98**	23 S	0R	30 S	25 S	10R	19I	23 S	0R	19IR	38 S	40 S	30 S
**LB100**	23 S	0R	26 S	20 S	10R	23 S	19IR	13R	17IR	24 S	30 S	20 S
**LB137**	21 S	0R	24 S	22 S	10R	22 S	20 S	15I	28 S	23 S	32 S	18I
**LB143**	27 S	0R	30 S	30 S	12R	22 S	23 S	0R	25 S	40 S	35 S	30 S
**LB166**	23 S	0R	25 S	25 S	0R	20 S	20 S	12R	23 S	30 S	25 S	23 S
**LB187**	18IR	10	30 S	32 S	12R	20 S	20 S	0R	22 S	32 S	30 S	25 S
**LB194**	25 S	0R	30 S	27 S	12R	20 S	22 S	0R	22 S	35 S	28 S	24 S
**LB195**	23 S	0R	29 S	27 S	12R	21 S	24 S	0R	20 S	36 S	38 S	30 S

Legend: Resistant (R) ≤ 14 mm; intermediate resistant (IR) 15–19 mm; susceptible (S) > 19 mm; AML (Amoxicillin); OX (Oxacillin); C (Chloramphenicol); PEN (Penicillin); CN (Cephalothin); TE (Tetracycline); KAN (Kanamycin); CIP (Ciprofloxacin); STR (Streptomycin); RAM (Rifampicin); IPM (Imipenem); GM (Gentamicin)

### Hemolytic activity

**Fig. 3:**
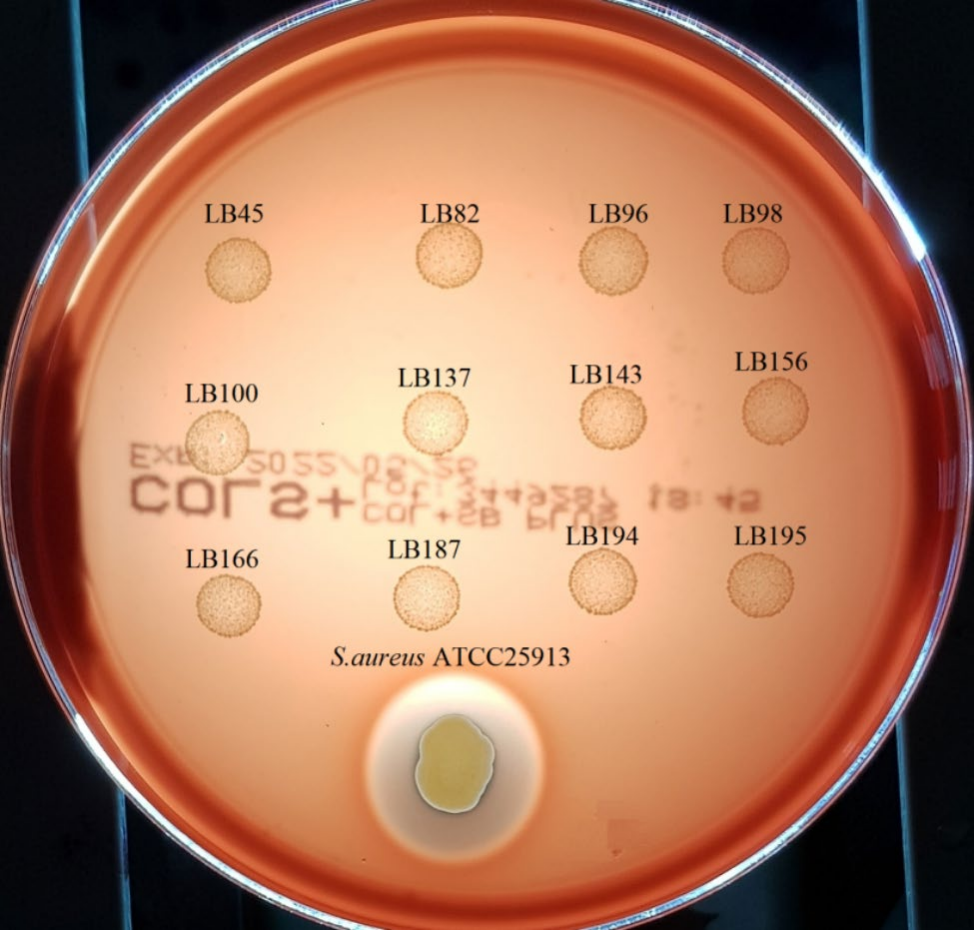
Hemolysis activity of LAB strains and *S. aureus* ATCC 25,913 (positive control)

Probiotic strains must be risk-free (γ-hemolysis), which makes them safe for consumption [15, 37]. In our study, all LAB strains showed γ-hemolysis activity (without clearing zones around the colonies on blood agar plates) (Fig. 3), thus ensuring the safety to be used as potential probiotics.

### Storage and preservation of LAB isolates: freeze-drying procedures

**Fig. 4:**
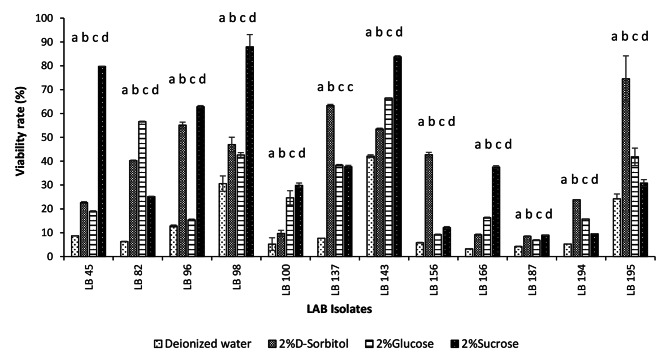
Viability rate of LAB isolates in the presence of different protective agents at end of freeze-drying Each value represented the mean value ± standard deviation (SD) (n = 3). Bars with different lower-case letters denoted significantly different (p < 0.05)

**Fig. 5:**
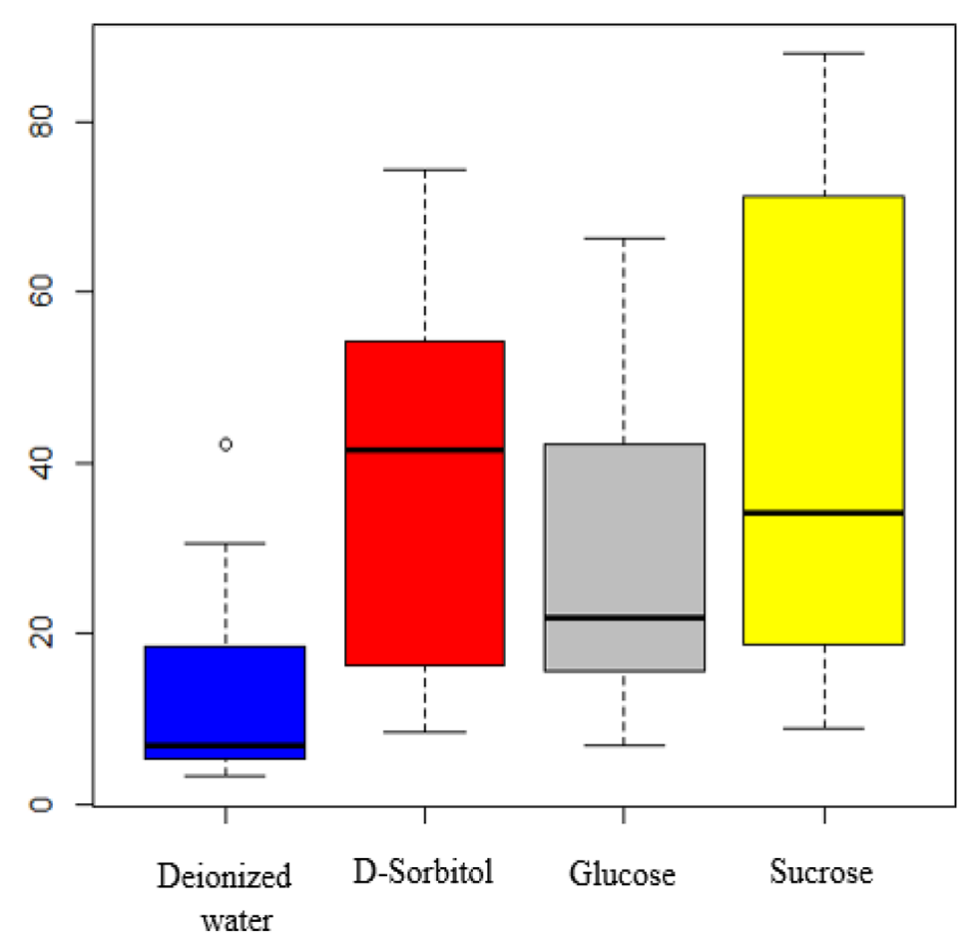
Affinity of LAB isolates for protective agents

Preservation of the viability of LAB strains during freeze-drying is a critical challenge. Three cryoprotectants (D-sorbitol, D-glucose, and sucrose) were tested for their ability to protect the LAB cells during freeze drying, while sterile deionized water served as the negative control. The survival rate of freeze-dried LAB isolates is presented in Fig. 4. A strain-dependent relationship existed between the cryoprotectant’s effectiveness. The isolates LB143 and LB98 had over 62% survival rate with all tested cryoprotectants, with maximum of 83.77 ± 0.44% and 87.97 ± 5.13% when sucrose was used as a cryoprotectant. On the contrary, the LB187 isolate had less than 10% survival rate with all cryoprotectants. LB143, LB96, LB137, and LB195 isolates demonstrated a high survival rate, from 53.50 ± 0.33% to 74.52 ± 9.64 with D-sorbitol (2%), whereas in the case of the LB82 and LB143 strains, glucose 2% (w/v) led to survival of 56.60 ± 0.18%, and 66.44 ± 0.14% respectively. Statistical analyses revealed a significant difference (p < 0.05) in the survival rates of different cryoprotectants between isolates. The boxplot presented in Fig. 5 shows that the D-sorbitol (41.48%) offered better protection of the LAB isolates during freeze-drying compared with sucrose (34.17%) and glucose (21.78%).

### Hierarchical ascending classification (HAC)

**Fig. 6:**
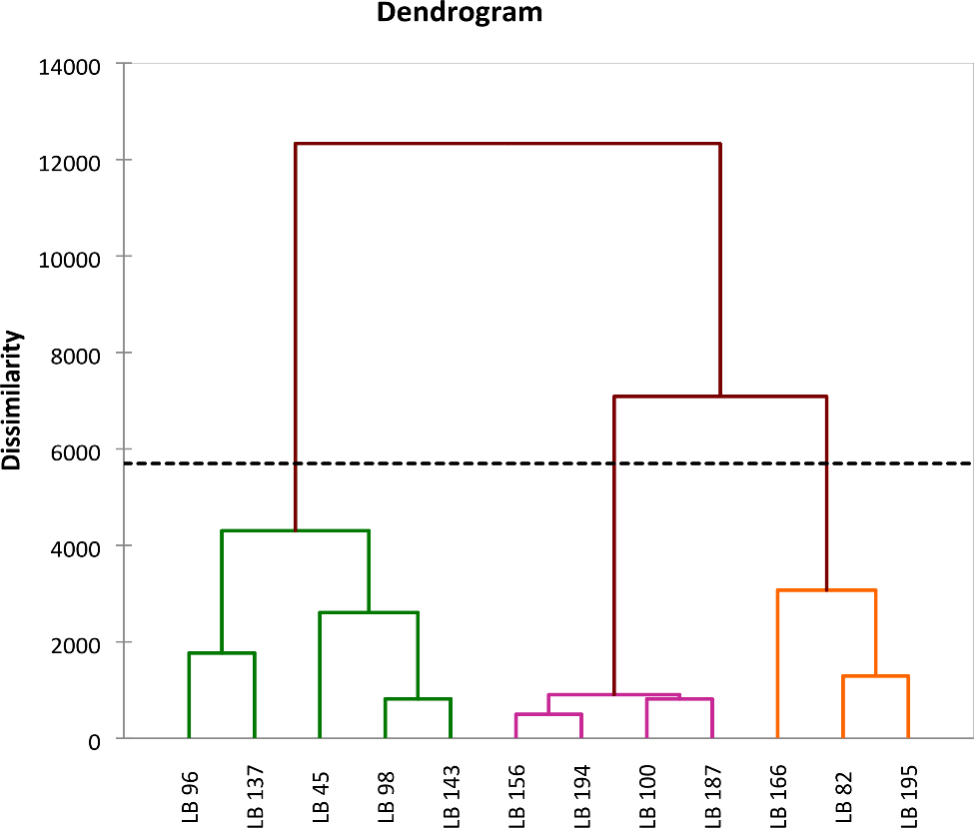
Cluster dendrogram of LAB species

The HAC carried out showed that all the LAB isolates had the same characteristics (Fig. 6). Three groups were generated, the first group included isolates LB 82, LB166, and LB 195. The second group included isolates LB194; LB187; LB156 and LB100. The last group consisted of isolates LB143; LB137; LB98; LB96, and LB45.

## Discussion

Today, aquaculture in the Ivory Coast has not yet reached a viable economic dimension, despite immense physical, hydrological (150 000 ha of lagoons, 350,000 ha of lakes, numerous shallows, etc.), climatic, and human resources [1]. The development of Ivorian aquaculture is hampered by several factors, the most significant of which are the availability of high-quality feed at exorbitant prices, a lack of technical skills, and the poor quality and quantity of fish [1]. Thus, the use of functional food (food enriched with probiotics) seems to be an ecological, economical, and sustainable solution.

Several aspects, including functional characteristics (antibacterial activity, biofilm-forming ability, simulation of gastrointestinal conditions, hydrophobicity, antioxidant activity), safety characteristics (molecular identification, antibiotic sensibility, and hemolytic activity), and storage (freeze-drying survival), have been taken into consideration in the selection process of LAB isolates as potential native probiotics.

Careful selection remains the main tool to obtain high quality probiotics. Proper strain identification at the species level is one key criterion to classify a microbial isolate as probiotics, especially for the microorganisms to be used in the food chain [74–76]. In this context, the well-known amplification and sequencing of the 16 S ribosomal DNA region are reliable tools to identify species at the expense of classical methods [74, 75]. In the present study, 12 LAB strains isolated from the intestine of Tilapia (*Oreochromis niloticus*) were identified by 16 S rDNA gene sequence homology analysis and belonged to *P. acidilactici* (9 strains), *P. pentosaceus* (2 strains), and *L. plantarum* (1 strain). The list of probiotics includes mainly members of the genera *Lactobacillus* and *Bifidobacterium* [35, 61, 71], but species belonging to the genus *Pediococcus*, particularly *P. acidilactici* and *P. pentosaceus*, have been investigated many times, indicating that newly isolated strains may play a key role in the new generation of functional ingredients [19, 77–80].

Some bacterial pathogens, such as *Escherichia sp., Klebsiella* sp., *Staphylococcus* sp., *Proteus* sp., and *Pseudomonas* sp., were isolated from fish and can indicate multiple sources of contamination [81–83]. All LAB isolates showed strong growth inhibition of all reference pathogens: *P. aeruginosa*, *E. coli*, *S. aureus*, *P. mirabilis*, and *K. pneumoniae*. These results are in agreement with previous studies where *Lactobacillus* and *Pediococcus* strains exhibited a broad spectrum of antagonistic activity against fish pathogens [19, 77, 84, 85].

In terms of probiotics, biofilm formation offered a more rapid capacity for metabolite production and resistance to hostile environments [47–50]. In our study, all the isolates showed a good ability to form biofilm. Lamari et al. [86] selected several LAB strains with the ability to form biofilm and high adherence to polystyrene microplates and hydrocarbon. This characteristic was important for bacteria’s ability to adhere to an abiotic surface, which could be a potential indicator for LAB to colonize the gut and further antagonize the pathogens [86].

Cell surface hydrophobicity is another property considered important for the probiotics’ overall adhesion capacity to various types of surfaces. The hydrophobicity of LAB isolates selected in the presence of three different solvents revealed that values depended on the solvent used. Overall, the hydrophobicity values of LAB isolates with chloroform were higher than those with other solvents. The values vary between 1.53% (LB 195) and 16.30% (LB 166) for hexane, 3.48% (LB 98) and 51.10% (LB 137) for xylene, and 9.4% (LB 96) and 87.2% (LB 166) for chloroform. Yasmin et al. [51] reported high hydrophobicity values for *Bifidobacterium* strains for xylene; in this work, the highest hydrophobicity values were obtained in the case of chloroform. The results are confirmed in another study on *L. fermentum* URLP18 and *L. lactis* URLA2 strains, which showed high aggregation capacities and high affinity towards xylene, followed by chloroform [84].

Free radical-scavenging ability, as a criterion for probiotics, had been studied by several authors [53, 54]. These authors reported that extracellular liquid, intracellular liquid, and intact cells had free radical-scavenging properties. In our study, CFS and intact cells were investigated. Remarkably, the CFS of the nine LAB strains exhibited higher DPPH scavenging activities than the intact cells, as follows: LB45 (90.23%); LB82 (51.92%); LB98 (74.29%); LB100 (84.06%); LB143 (75.57%); LB156 (64.52%); LB166 (63.85%); LB187 (57.84%); and LB194 (76.60%). Yasmin et al. [51] reported that the *Bifidobacterium* exhibited strong antioxidant activity in cell-free supernatant, whose values varied between 80.72% and 87.72%.

Several studies reported the ability of probiotic microorganisms to produce extracellular enzymes improving the nutrient digestibility, growth performances, and health status of fish [13, 36, 55, 56]. For instance, Iorizzo et al. [36] showed that the LABs isolated from the intestinal tract of the Mediterranean trout (*Salmo macrostigma*) are producers of extracellular enzymes that help absorb the nutrients in the fish intestine. Our results showed the ability of 9 out of 12 LAB isolates to synthesize both β-galactosidase and lipases. No other enzymatic activity (amylases, cellulases, and proteases) was detected. This finding was in contrast with the results reported by Muñoz-Atienza et al. [42], in which the majority of the LAB strains did not shown lipolytic activity (with few exceptions). On the other hand, the LAB strains isolated from the intestine of freshwater fish species exhibited amylase, lipase, and protease activities [84]. Similarly, Marchwiska and Gwiazdowska [87] reported that different *Lactobacillus* and *Pediococcus* isolated from swine faeces for feed additive composition had protease and amylase activities, but no lipase activity. *Bacillus* species have already been known as enzyme producers, being one of the reasons for their use in aquaculture as probiotics enhancing feed digestibility, digestive enzyme activities, and growth performance [21].

Furthermore, antibiotic susceptibility is a prerequisite from a safety standpoint because probiotic bacteria might transfer antibiotic-resistance genes either directly or indirectly to pathogenic bacteria. This approach requires evidence that the LAB strain does not show resistance to antibiotics used in human and veterinary medicine. In our study, all LAB isolates were susceptible or intermediately resistant to 9 antibiotics and resistant to 3 antibiotics out of the 12 antibiotics used. LAB isolates showed resistance to oxacillin, gentamycin, and ciprofloxacin. Our result was important because LAB isolates showed sensitivity to penicillin, ampicillin, and chloramphenicol, among the most commonly used antibiotics in aquaculture. The results obtained were not consistent with those documented by Diguță et al. [78], which indicated that two *Pediococcus* strains (L3 and L5) isolated from the Kombucha consortium were found to be resistant to amoxicillin, streptomycin, kanamycin, and tetracycline. Furthermore, antibiotic sensitivity is a variable and strain-dependent property. In the context of probiotic LAB selection, drug-resistant probiotic bacteria are serious health threats. Increasing and abusive use of antibiotics has given rise to resistant bacteria through the transfer of resistance plasmids between bacteria [7–9, 88–90]. The resistance to an antibiotic may be accompanied by resistance to one or more other antibiotics, even if the bacteria have no contact with these antibiotics. Bacterial strains with transferable antibiotic resistance genes should not be used in animal feeds, fermentation, or probiotic foods for human consumption, according to the European Food Safety Authority (EFSA) [91]. Given the increase of drug-resistant probiotics has been recently developed the online ProbResist database, which centralizes reports of probiotic bacteria that have been demonstrated experimentally to be resistant to antibiotics [92].

The examination of hemolytic activity is strongly recommended by the EFSA as long as the isolated bacteria are intended for use in food products, even if they have “generally recognized as safe” (GRAS) or “quality presumption of safety” (QPS) status. In this study, all the isolates exhibited no hemolytic activity (γ-hemolysis), indicating that they are non-pathogenic and considered safe for animal or human probiotic applications. Similar results were previously obtained with two *Pediococcus* strains isolated from Kombucha (L3 and L5) [78]. Yasmin et al. [51] shown that eight *Bifidobacterium* strains isolated from raw camel milk did not exhibit hemolytic activity. Lack of hemolytic activity is significant during the selection of probiotic strains when it comes to probiotic safety because such strains are non-virulent, and the lack of hemolysin assures that virulence will not arise among bacterial strains [15].

The ability of LAB strains to effectively function in the gastrointestinal tract (including bile salts tolerance and low gastric pH resistance) is the most important criterion for their selection as probiotics. Probiotic bacteria must first make it through the stomach, where the pH can be as low as 1.5 to 2 before reaching the intestinal tract [93, 94]. In our study, the resistance tests of these bacteria at low pH levels (ranging from 1 to 3) revealed that all strains are resistant at pH 1.5 for 3 h, while most strains lose viability in 1 h at pH 1.5. The resistance at 0.3% pepsin and low pH (1.5) was characterized by a high level of cell viability. The survival rates of the 12 LAB isolates ranged from 34.8 to 49.9%. Chemlal-Kerrhaz et al. [39] showed that two isolates of LAB from the Nile Tilapia (*Oreochromis niloticus*) tolerated a concentration of 0.3% bile salts for 4 h and pH 2 for 3 h.

In addition, all strains tolerated the concentration of 0.3% bile salts for 4 h. The growth rate of the 12 LAB isolates varied between 0.92 and 21.46%. The results obtained were lower than in previous studies which reported high survival abilities of different *Pediococcus* strains [78] and *L. fermentum* URLP18 isolated from *C. mrigala* [84] in the presence of high bile salts concentration (until 2%). Our study showed a similar outcome; all isolates tolerated a concentration of 0.3% bile salts for 4 h and exhibited resistance at pH 1.5, however with different intensities.

Freeze-drying, as a LAB conditioning technique, is recognized to cause severe damage to organisms, particularly at the membrane level as well as to their proteins, but the addition of cryoprotective agents may mitigate injury or inactivation by increasing cell survival during freeze-drying [78, 95]. In this study, three cryoprotective agents were assessed for their influence on the LAB isolates’ viability rate at the end of the freeze-drying process. Our results indicate that the LAB isolates showed an important affinity for D-sorbitol and sucrose. This trend has been observed by Diguță et al. [78], where sucrose was responsible for the best viability rate of *P. acidilactici* and *P. pentosaceus* at the end of freeze-drying. Considering the strain-dependent variation in response to the stress conditions during the freeze-drying process, cryoprotectant agents must be investigated to choose them for conditioning of LAB strains with high efficiency on cell viability and economically feasible, before being included in functional foods or feeds.

## Conclusion

Based on 16 S rDNA gene sequencing, 12 LAB strains isolated from the intestine of Tilapia (*Oreochromis niloticus*) were identified as belonging to *P. acidilactici, P. pentosaceus*, and *L. plantarum* species with a predominance of *P. acidilactici*. All LAB isolates showed a high antibacterial activity as well as a strong antioxidant activity. Additionally, they showed no hemolytic activity, a typical pattern of antibiotic susceptibility, and a good ability to form a biofilm, respectively. All LAB isolates exhibited either lipase or β-galactosidase or both enzymes production. Some LAB strains showed good survival rates in the simulated gastrointestinal conditions. Conditioning of LAB strains by freeze-drying using D-sorbitol or sucrose as cryoprotectant agents could be used to formulate probiotic products in powdered form. According to these results, *P. acidilactici* LB137 and *P. pentosaceus* LB195 present promising probiotic properties and could be applied as health promoters for fish. Further in vivo studies might use these strains in monoculture or co-culture to obtain enriched/supplemented food for fish farming, ultimately ensuring that healthier fish will be part of a healthier human diet.

## Materials and methods

### Bacterial strains and culture conditions

Twelve LAB strains were isolated from the intestine of Tilapia (*Oreochromis niloticus*) originating from the aquaculture farm of Oceanologic Research Center in Ivory Coast. Five pathogenic bacteria including *P. mirabilis* JCM1669 (University Nangui Abrogoua of Ivory Coast), *P. aeruginosa* ATCC 27853, *E. coli* ATCC 25922, *K. pneumoniae* ATCC 43816, and *S. aureus* ATCC 25913 (American Type Culture Collection (ATCC), Manassas, VA, USA) were used as indicator strains. LAB strains were routinely grown in MRS (De Man, Rogosa, and Sharpe) broth or agar (Oxoid Limited, Hampshire, United Kingdom) for 24–48 h at 37 °C under microaerophilic conditions (5% CO_2_). The reference pathogenic bacteria were grown in tryptic soy broth (TSB) or agar (TSA) (Scharlab S.L., Barcelona, Spain) for 18–24 h at 37 °C under aerobic conditions. All strains were stored at − 20 °C in an adequate culture medium containing 30% (*v*/*v*) glycerol (Scharlab S.L., Barcelona, Spain) and subcultured twice before being used in assays.

### Identification of LAB strains

LAB strains were grown in MRS broth for 48 h at 37 °C. Cells were harvested by centrifugation at 5000 x g for 10 min. Genomic DNA extraction was performed using a ZR Fungal/Bacterial DNA kit (Zymo Research, Irvine, CA, USA), according to the manufacturer’s instructions. The DNA concentration and purity were verified with a SpectraMax® QuickDrop™ (Molecular Devices, San Jose, CA, USA). LAB strains were identified by analysis of 16 S rDNA amplified with the universal primers 27 F (AGAGTTTGATCMTGGCTCAG) and 1492R (TACGGYTACCTTGTTACGACTT) (Biolegio B.V. Nijmegen, The Netherlands). The reaction mixture consisted of 50 µl of 10X DreamTaq Green Buffer (contains 20 mM MgCl_2_), 0.5 µM of each primer, 0.2 mM dNTPs, 0.025 U of DreamTaq DNA Polymerase (Thermo Fisher Scientific, Baltics, UAB, Vilnius, Lithuania), and 10 ng of bacterial DNA. The amplification program cycles started with an initial denaturation at 95 °C for 3 min, followed by 35 cycles (94 °C for 1 min, 55 °C for 1 min, 72 °C for 1 min), and a final extension step at 72 °C for 7 min. The amplification reactions of the 16 S rDNA region were performed using a thermal cycler (MultiGene Thermal Cycler Labnet International, Inc., Edison, NJ, USA). The PCR products were detected by agarose gel electrophoresis (2% (w/v) agarose, 90 V, 60 min), and visualized using a GelDoc-It Imaging System (Analytik Jena, Upland, CA, USA). Sequencing was performed in both directions with the universal primers 27 F and 1492R by Cellular and Molecular Immunological Application, Holland (CEMIA, Greece). The partially obtained nucleotide sequences were aligned with multiple available homologous sequences in the National Center for Biotechnology Information (NCBI) GenBank databases (http://www.ncbi.nlm.nih.gov, accessed on 29 November 2021) to identify at the species level based on high similarity. The phylogenetic tree was constructed via the neighbour-joining method [96] using MEGA (Molecular Evolution Genetic Analysis) software, version X [97].

### Functional characterization LAB isolates

#### Antibacterial activity of LAB isolates

Antagonistic activities of the LAB isolates were recorded against five pathogens indicators (*E. coli* ATCC 25922, *K. pneumoniae* ATCC 43816, *P. aeruginosa* ATCC 27853, *P. mirabilis* JCM1669, and *S. aureus* ATCC 25913) by the agar well diffusion method described by Balouiri et al. [98] with some modifications. The LAB isolates were cultured in MRS broth at 37 °C for 48 h. and centrifuged at 10,000 x g at 4 °C, for 5 min. Cell-free supernatants (CFS) were obtained by filtration using sterile 0.22 μm Millipore filters (VWR International, Rosny-sous-Bois, France). 1mL of the overnight pathogen culture (adjusted OD_600 nm_ to 0.2 ± 0.05, representing approximately 10^7^− 10^8^ cfu/ml) was added to a sterile Petri dish (90 mm), overlaid with approximately 20 mL of TSA cooled to 45 °C, and gently homogenized until solidification. Wells with a diameter of 6 mm have been punched aseptically with a sterile tip, filled with 100 µl of CFS tested, and incubated at 37 °C for 24 h. A clear zone of 1 mm or more around each well was considered positive inhibition, which demonstrated the antibacterial activity of the CFS.

### Ability to form a biofilm

The LAB isolates were grown in MRS broth at 37 °C for 48 h and bacterial load was adjusted to the same optical density OD_600 nm_ of 0.2 ± 0.05. Bacterial cells were centrifuged at 4000 rpm for 10 min and the pellets were washed three times with NaCl solution (0.9%) and dried at 50 °C for 30 min. The bacterial biofilms were stained with 1 ml of 0.1% crystal violet (Sigma Aldrich, Saint Louis, MO, USA) for 20 min and washed with the NaCl solution until the liquid was clear. The dye was eluted with ethanol (96%). The quantification was performed by measuring absorbance value (OD) at the 595 nm wavelength spectrophotometer (BioBase, Jinan, Shandong, China). The ability to form a biofilm was considered positive for OD ≥ 0.5.

### Tolerance of LAB isolates to bile salts

The bile salts tolerance test of the LAB isolates was performed according to the method described by Diguță et al. [78] with some modifications. Test tubes containing MRS broth were supplemented with 0.3% bile salts (Oxoid Limited, Hampshire, United Kingdom), inoculated with each LAB isolate (adjusted to the OD_600 nm_ at 0.2 ± 0.05), and incubated at 37 °C, for 4 h. The cell viability was tested by the plate count method at 0 h and after 4 h of incubation. Tolerance toward bile salts was estimated by$\text{g}\text{r}\text{o}\text{w}\text{t}\text{h} \text{r}\text{a}\text{t}\text{e}=\left(\frac{\text{l}\text{o}\text{g} \text{C}\text{F}\text{U} \text{N}\text{i} }{\text{l}\text{o}\text{g} \text{C}\text{F}\text{U} \text{N}\text{t}}\right)\times 100$, where Ni and Nt mean the viable cells (CFU/ml) at 0 h and after 4 h of incubation.

### Resistance of LAB isolates to pepsin and acid pH

The ability of LAB isolates to survive the presence of pepsin and acid pH was done using the method described by Diguță et al. [78]. After overnight culture, LAB cells were centrifuged at 2000 x g for 10 min and pellets were suspended and washed twice with sterile physiological saline (0.9% NaCl). The pellets were suspended in phosphate-buffered saline (PBS) solution (VWR International, Rosny-sous-Bois, France) previously supplemented with 0.3% pepsin (Sigma-Aldrich, Saint Louis, MO, USA) and pH was adjusted to 1.5 with 1 N HCl. The cell viability was tested by the plate count method at 0 h and after 3 h of incubation. The percentage (%) survival of LAB isolates was calculated by the following formula: $\% \text{v}\text{i}\text{a}\text{b}\text{i}\text{l}\text{i}\text{t}\text{y}=\left(\frac{\text{l}\text{o}\text{g} \text{U}\text{F}\text{C} \text{N}\text{t} }{\text{l}\text{o}\text{g} \text{U}\text{F}\text{C} \text{N}\text{i}}\right)\times 100$, where Ni and Nt mean the viable cells (CFU/ml) at 0 h and after 3 h of incubation.

### Evaluation of LAB isolates hydrophobicity

Overnight LAB cultures were centrifuged at 12000 x g for 5 min at 4 °C. The cell pellets were washed twice using PBS solution (pH 7.2) and adjusted to the optical density of 1 ± 0.05 at 650 nm wavelength (H_0_). To determine the cell surface hydrophobicity, three solvents were used: hexane (VWR International, Rosny-sous-Bois, France), xylene (Bernd Kraft GmbH, Duisburg, Germany), and chloroform (Bernd Kraft GmbH, Duisburg, Germany). The mixture of 2.4 ml of cell suspension with 0.4 mL of solvent was vigorously vortexed for 2 min. After the phase stabilization and separation period of 30 min at room temperature, the aqueous phase was carefully recovered and the optical density was measured at 650 nm wavelength (H_1_). The hydrophobicity values were calculated according to the following formula: $H \%=\left(\frac{\text{H}0 - \text{H}1}{\text{H}0}\right)\times 100$.

### DPPH Free Radical Scavenging ability

After the LAB cells were incubated at 37 °C in MRS broth overnight, the cells were harvested by centrifugation at 12000 x g for 5 min at 4 °C. The supernatant samples were collected and cell pellets were washed twice with PBS solution and suspended in the same solution to adjust to OD_600 nm_ 0.2 ± 0.05 and served as intact cells. The 1-diphenyl-2-picrylhydrazyl (DPPH) scavenging activity was determined by the method described by Brand-Williams et al. [99]. A volume of 2 ml of DPPH (Alfa Aesar, Kandel, Germany) (100 µM in methanol) was added to 1 ml of the cell suspension or 1 ml of the supernatant, the mixtures were mixed vigorously and incubated at laboratory temperature in the dark for 30 min. In the case of the intact cell, the absorbance of the resulting solution was measured in triplicate at 517 nm wavelength after centrifugation at 12,000 x g, for 5 min. The deionized water was used as the negative control. The presence of antioxidant activity is shown by the change in color of the mixture from purple to yellow. The scavenging ability was defined as: $\% \text{A}\text{A}=\left(\frac{\text{O}\text{D} \text{D}\text{P}\text{P}\text{H}-\text{O}\text{D} \text{s}\text{a}\text{m}\text{p}\text{l}\text{e}}{\text{O}\text{D}\text{D}\text{P}\text{P}\text{H}}\right)\times 100$.

### Plate screening of enzymes producing LAB isolates

LAB isolates were inoculated in spots on the surface of culture media distributed in Petri dishes. Amylase activity was evaluated on MRS Agar medium supplemented with 1% of soluble starch (VWR International, Rosny-sous-Bois, France). After incubation at 37 °C for 4 days, the positive reaction was indicated by a clear zone surrounding LAB isolates by adding Iodine-potassium iodide solution (Carl Roth GmbH & Co. KG, Karlsruhe, Germany). Cellulase activity was tested on MRS Agar supplemented with 1% carboxymethylcellulose (Sigma-Aldrich, Merck, Darmstadt, Germany). The zone of clearance was visualized after staining with 0.1% Congo red solution (Sigma-Aldrich, Merck, Darmstadt, Germany) and washing the plate with 1 M NaCl. Lipase activity was determined on MRS Agar medium supplemented with 0.25 mL olive oil, 0.01% CaCl_2_xH_2_O, and 0.0001% (w/v) rhodamine B (Alfa Aesar, Kandel, Germany). Positive reactions were observed by pink-orange colony under UV 350 nm. Protease activity was detected on skim milk (1%) agar medium (PanReac AppliChem, Darmstadt, Germany). After the incubation period, the LAB isolates showing a clear zone of the degradation of casein were read as positive for protease production. The β-galactosidase activity was determined on MRS agar containing 20 µl of X-Gal (20 mg/ml in DMSO) (PanReac AppliChem, Darmstadt, Germany). The green color colonies were regarded as bacteria producing β-galactosidase enzyme.

### Safety characterization of the LAB isolates

#### Antibiotic susceptibility

The LAB strains were tested for antibiotic susceptibilities by the disc diffusion method described by CLSI [100]. Twelve (12) antibiotics belonging to 8 classes of antibiotics were used, namely Beta-lactams (Penicillin: PEN 6 µg; Amoxicillin: AML 10 µg; Oxacillin: OX 5 µg), Cephalosporins (Cephalothin: CN 30 µg), Aminoglycosides (Gentamicin: GM 10 µg; Kanamycin: KAN 1 mg; Streptomycin: STR 500 µg), Quinolones (Ciprofloxacin: CIP 5 µg), Cyclines (Tetracycline: TE 30 µg), Rifampicin (Rifampicin: RAM 30 µg), Carbapenems (Imipenem: IPM 10 µg), Phenicols (Chloramphenicol: C 30 µg). All antibiotics were provided by Oxoid Limited (Hampshire, United Kingdom). A volume of 100 µl of LAB fresh cultures (adjusted to the OD_600 nm_ at 0.2 ± 0.05) was inoculated into MRS agar plates and dried. Antibiotic discs were placed on the MRS plates agar and incubated at 37 °C, for 48 h. The diameter of the zone of inhibition was measured and classified as sensitive (S); intermediate resistant (IR); or resistant (R) in agreement with the Clinical and Laboratory Standards Institute CLSI [100].

### Hemolytic activity of LABs

An aliquot of each LAB culture (5µL) was applied onto Columbia Agar plates containing 5% (w/v) sheep blood (Oxoid Limited, Hampshire, United Kingdom) and cultured at 37 °C, for 48 h. The hemolytic activity was assessed by β-hemolysis (the clear halo around colonies), α-hemolysis (the green halo around colonies), or γ-hemolysis (no halo around colonies). Here, *S. aureus* ATCC 25913 (β-hemolytic) was used as a positive control strain.

### Storage and preservation of LAB isolates: freeze-drying procedures

The LAB cultures left in MRS broth overnight were centrifuged at 4000 x g for 10 min at 4 °C. The pellets were washed twice using saline solution (0.9% NaCl) and suspended in 2 ml of cryoprotectant solutions. Three cryoprotectants (at a final concentration of 2%) were tested: D-sorbitol (Sigma-Aldrich, Saint Louis, MO, USA), D-glucose, and sucrose (PanReac AppliChem, Darmstadt, Germany). After freezing at -20 °C overnight, cell suspensions (prepared as described above) were freeze-dried in a chamber-type freeze-dryer (FreeZone6, LABCONCO, 6 L Benchtop Freeze Dry System, Kansas, MO, USA) at − 55 °C and 0.3 mbar, for 4 h. The cell viability was tested before and after the freeze-drying procedure by the plate count method. Distilled water was used as a control. The survival rate of LAB strains was calculated as:

$\text{\%} \text{v}\text{i}\text{a}\text{b}\text{i}\text{l}\text{i}\text{t}\text{y}=\left(\frac{\text{l}\text{o}\text{g} \text{C}\text{F}\text{U} \text{N} }{\text{l}\text{o}\text{g} \text{C}\text{F}\text{U} \text{N}0}\right)\times 100$, where N_0_ and N mean the viable cells (CFU/ml) before and after freeze drying, respectively.

### Statistical analysis

All the experiments were carried out in triplicate and the results were expressed as the mean ± standard deviation. The calculations, figures, and boxplots were performed using Excel 2016. For the comparison of the means of the studied parameters, one-way analysis of variance (ANOVA) and Tukey’s test were performed with the XLStat software (Version 2016). For p < 0.05, the means were considered significant. XLSTAT software was used to create a dendrogram to group LAB species with similar characteristics.

## Data Availability

All the datasets generated in the present study are included in the manuscript. The partial 16 S rDNA sequences of LAB strains were deposited in the NCBI GenBank. Accession numbers: https://www.ncbi.nlm.nih.gov/nuccore/ON141894.1/. https://www.ncbi.nlm.nih.gov/nuccore/ON141895.1/. https://www.ncbi.nlm.nih.gov/nuccore/ON141896.1/. https://www.ncbi.nlm.nih.gov/nuccore/ON141897.1/. https://www.ncbi.nlm.nih.gov/nuccore/ON141898.1/. https://www.ncbi.nlm.nih.gov/nuccore/ON141899.1/. https://www.ncbi.nlm.nih.gov/nuccore/ON141900.1/. https://www.ncbi.nlm.nih.gov/nuccore/ON141901.1/. https://www.ncbi.nlm.nih.gov/nuccore/ON141902.1/. https://www.ncbi.nlm.nih.gov/nuccore/ON141903.1/. https://www.ncbi.nlm.nih.gov/nuccore/ON141904.1/. https://www.ncbi.nlm.nih.gov/nuccore/ON141905.1/.
